# Crystal structure and Hirshfeld surface analysis of 4-{[(anthracen-9-yl)meth­yl]amino}­benzoic acid di­methyl­formamide monosolvate

**DOI:** 10.1107/S2056989020005393

**Published:** 2020-04-24

**Authors:** Adeeba Ahmed, Aiman Ahmad, Musheer Ahmad, Valentina A. Kalibabchuk

**Affiliations:** a Department of Applied Chemistry, ZHCET, Aligarh Muslim University, Aligarh 202002 (UP), India; b Department of General Chemistry, O. O. Bohomolets National Medical University, Shevchenko Blvd. 13, 01601 Kiev, Ukraine

**Keywords:** crystal structure, 4-amino­benzoic acid (PABA), anthracene, inter­molecular hydrogen bonding, C—H⋯π inter­actions, Hirshfeld surface analysis

## Abstract

The title compound, C_22_H_17_NO_2_·C_3_H_7_NO, was synthesized by condensation of an aromatic aldehyde with a secondary amine and subsequent reduction. It was crystallized from a di­methyl­formamide solution as a monosolvate, C_22_H_17_NO_2_·C_3_H_7_NO. The aromatic mol­ecule is non-planar with a dihedral angle between the mean planes of the aniline moiety and the methyl anthracene moiety of 81.36 (8)°. The torsion angle of the C_ar­yl_—CH_2_—NH—C_ar­yl_ backbone is 175.9 (2)°. The crystal structure exhibits a three-dimensional supra­molecular network, resulting from hydrogen-bonding inter­actions between the carb­oxy­lic OH group and the solvent O atom as well as between the amine functionality and the O atom of the carb­oxy­lic group and additional C—H⋯π inter­actions. Hirshfeld surface analysis was performed to qu­antify the inter­molecular inter­actions.

## Abstract 2

In the crystal structure of the title compound inter­molecular hydrogen-bonding inter­actions and weak C—H⋯π inter­actions between the constituents lead to the formation of a three-dimensional network. Hirshfeld surface analysis revealed that H⋯H inter­actions dominate the crystal packing.

## Chemical context   

Schiff bases belong to a class of organic compounds that are formed by the condensation reaction of a carbonyl carbon with an aliphatic/aromatic amine, resulting in the formation of a characteristic imine bond (–HC=N–). Many Schiff bases exhibit activities of biological and pharmaceutical significance. Moreover, Schiff bases are actively used as organic linkers for building metal complexes with inter­esting properties.
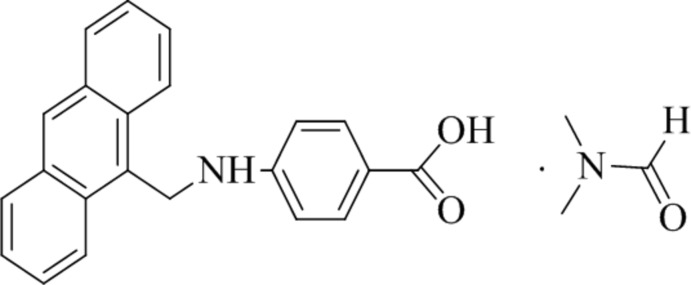


Here we report the synthesis and crystal structure of a reduced Schiff base that was formed by a condensation reaction of anthraldehyde with 4-amino benzoic acid (PABA). The title compound crystallizes with a di­methyl­formamide (DMF) solvent mol­ecule in a 1:1: ratio. Both anthraldehyde and PABA have shown anti­cancer (Pavitha *et al.*, 2017 ▸), fluorescence (Obali & Ucan, 2012 ▸; Singh *et al.*, 2014 ▸), sensing (Zhou *et al.*, 2012 ▸ *)*, anti­microbial (Vidya, 2016 ▸) and magnetic properties (Dianu *et al.*, 2010 ▸).

## Structural commentary   

**Figure 1:**
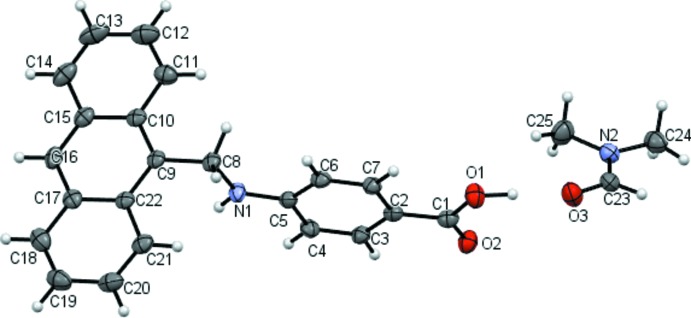
The mol­ecular structures of the components in the title compound. Displacement ellipsoids are drawn at the 50% probability level.

The title mol­ecule is non-planar, with the tricyclic fragment nearly perpendicular to the phenyl ring of the PABA moiety, making a dihedral angle of 81.36 (8)° (Fig. 1 ▸). The torsion angle of the C_ar­yl_—CH_2_—NH—C_ar­yl_ backbone (C9—C8—N1—C5) is 175.9 (2)°. The C8—N1 bond length of 1.452 (3) Å is in agreement with the corresponding bond length of 1.457 (3) Å in the solvent-free compound [CSD (Groom *et al.*, 2016 ▸) refcode RUCJIL; Ahmed *et al.*, 2020 ▸], just as the bond lengths in the carb­oxy­lic group of the title compound, C1—O2 = 1.230 (3), C1—O1 = 1.322 (3) Å, are virtually identical with those of the solvent-free compound [1.238 (3) and 1.325 (3) Å, respectively].

## Supra­molecular features   

**Figure 2:**
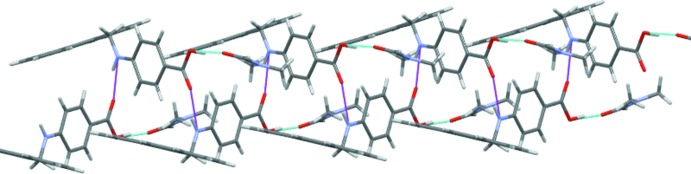
View along [010] showing a layer formed by hydrogen-bonding inter­actions between the mol­ecule and the solvent. Purple and blue dashed lines represent the N—H⋯O and O—H⋯O bonds, respectively.

**Figure 3:**
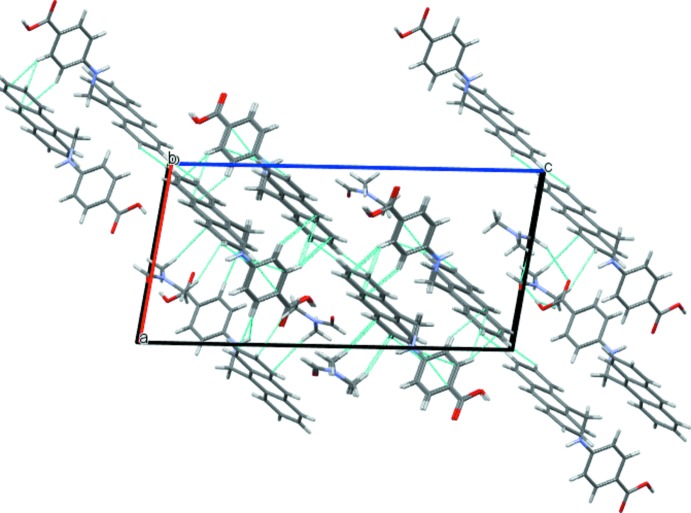
The crystal packing showing C—H⋯π inter­actions between the layers, building up a three-dimensional network.

Classical hydrogen-bonding inter­actions between the carb­oxy­lic OH group (O1) and the solvent O atom (O3) as well as between the amine functionality (N1) and the O atom of the carb­oxy­lic group (O2) lead to the formation of supra­molecular layers extending parallel to (10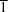) (Fig. 2 ▸, Table 1 ▸). C—H⋯π inter­actions involving the phenyl C—H groups of PABA as donor groups and the π system of the anthracene moiety link adjacent layers into a three-dimensional network (Fig. 3 ▸, Table 1 ▸).

## Hirshfeld Surface Analysis   

**Figure 4:**
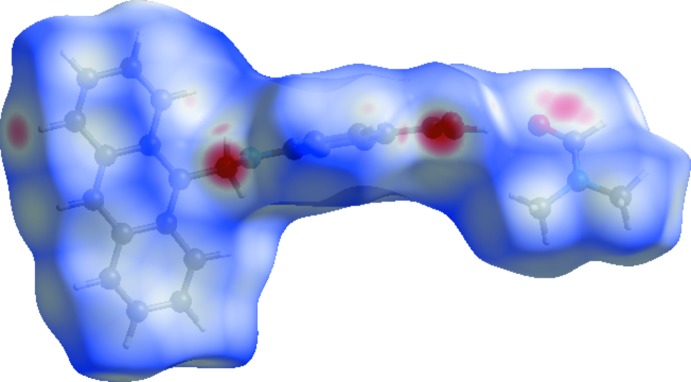
Hirshfeld surface of the two mol­ecules in the title compound mapped over *d*_norm_, in the colour range −0. 461 to 1.471 a.u..

Hirshfeld surface analysis (Spackman & Jayatilaka, 2009 ▸) and the associated two-dimensional fingerprint plots (McKinnon *et al.*, 2007 ▸) were performed with *CrystalExplorer* (Turner *et al.*, 2017 ▸). The Hirshfeld surfaces are colour-mapped with the normalized contact distance, *d*_norm_, varying from red (distances shorter than the sum of the van der Waals radii) through white to blue (distances longer than the sum of the van der Waals radii). The positions of the O—H⋯O and N—H⋯O hydrogen bonds between the mol­ecules are indicated by the red regions on the Hirshfeld surface (Fig. 4 ▸).

**Figure 5:**
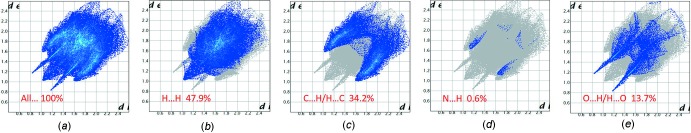
(*a*) Two-dimensional fingerprint plot of the title compound, and those delineated into (*b*) H⋯H, (*c*) C⋯H/H⋯C, (*d*) N⋯H/H⋯N and (*e*) O⋯H/H⋯O inter­actions.

The two-dimensional fingerprint plot (Fig. 5 ▸ *a*) and those delineated into (*b*) H⋯H, (*c*) C⋯H/H⋯C, (*d*) N⋯H/H⋯N and (*e*) O⋯H/H⋯O inter­actions reveal contributions of 47.9%, 34.2%, 0.6% and 13.7%, respectively, to the overall surface.

## Database survey   

Next to the solvent-free crystal structure (RUCJIL; Ahmed *et al.*, 2020 ▸), a search of the Cambridge Structural Database (CSD,Version 5.40, update August 2019; Groom *et al.*, 2016 ▸) for the *N*-(anthracen-9-ylmeth­yl)aniline skeleton gave six hits, five polymeric metal complexes of the ligand 5-[(anthracen-9-ylmeth­yl)amino]­isophthalic acid containing gadolinium (VOLSOG, VOLSUM, VOLTAT, VOLTIB; Singh *et al.*, 2014 ▸) and cadmium (EYUMOC; Yan *et al.*, 2016 ▸) as well as an organic mol­ecule with a calix(4)arene ring (Bu *et al.*, 2004 ▸). In these structures, the bridging C—N bond length varies from ≃ 1.389 to 1.494 Å, compared to the C8—N1 bond length of 1.452 (3) Å in the title structure.

## Synthesis and crystallization   

The Schiff base was synthesized and subsequently reduced by a reported procedure (Ahmed *et al.*, 2020 ▸). To this reduced ligand (0.15 mmol), ethanol and di­methyl­formamide were added in an equal volume ratio, and the mixture was heated under reflux for 3–4 h at 353 K. The solution was then allowed to cool to room temperature, filtered and kept for slow evaporation. After 10 to 12 d, small colourless block-like crystals began to grow that were dried and characterized by single crystal X-ray diffraction.

## Refinement   

Crystal data, data collection and structure refinement details are summarized in Table 2 ▸. Hydrogen atoms bound to N or O atoms were located in a difference-Fourier map and were freely refined, while the C-bound hydrogen atoms were included in calculated positions and allowed to ride on their parent C atom: C—H = 0.93–0.97 Å with *U*_iso_(H) = 1.2*U*_eq_(C).

## Supplementary Material

Crystal structure: contains datablock(s) I. DOI: 10.1107/S2056989020005393/wm5548sup1.cif

Structure factors: contains datablock(s) I. DOI: 10.1107/S2056989020005393/wm5548Isup2.hkl

CCDC reference: 1982147

Additional supporting information:  crystallographic information; 3D view; checkCIF report

## Figures and Tables

**Table 1:** Hydrogen-bond geometry (Å, °) *Cg*5 and *Cg*7 are the centroids of the 10-membered ring system C9–C22 and of the 14-membered anthracene moiety, respectively.

*D*—H⋯*A*	*D*—H	H⋯*A*	*D*⋯*A*	*D*—H⋯*A*
O1—H1⋯O3	1.02 (4)	1.59 (4)	2.590 (3)	167 (4)
N1—H1A⋯O2^i^	0.88 (1)	2.13 (1)	2.973 (3)	160 (1)
C18—H18⋯O3^ii^	0.95 (1)	2.40 (1)	3.277 (4)	154 (1)
C6—H6⋯*Cg*7^iii^	0.95	2.80 (1)	3.552 (2)	137 (1)
C7—H7⋯*Cg*5^iii^	0.95	2.99 (1)	3.646 (3)	138 (1)

Symmetry codes: (i) 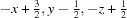; (ii) 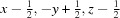; (iii) 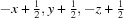.

**Table 2:** Experimental details

Crystal data	
Chemical formula	C_22_H_17_NO_2_·C_3_H_7_NO
*M*_r_	400.48
Crystal system, space group	Monoclinic, *P*2_1_/*n*
Temperature (K)	100
*a*, *b*, *c* (Å)	10.6878 (9), 8.9088 (7), 21.9503 (19)
β (°)	99.049 (3)
*V* (Å^3^)	2064.0 (3)
*Z*	4
Radiation type	Mo *K*α
μ (mm^−1^)	0.09
Crystal size (mm)	0.36 × 0.28 × 0.16

Data collection	
Diffractometer	Bruker APEXII CCD
Absorption correction	Multi-scan (*SADABS*; Bruker, 2016 ▸)
*T*_min_, *T*_max_	0.368, 0.746
No. of measured, independent and observed [*I* ≥ 2u(*I*)] reflections	31593, 3668, 2477
*R*_int_	0.139
(sin θ/λ)_max_ (Å^−1^)	0.596

Refinement	
*R*[*F* ^2^ > 2σ(*F* ^2^)], *wR*(*F* ^2^), *S*	0.057, 0.184, 1.12
No. of reflections	3668
No. of parameters	278
H-atom treatment	H atoms treated by a mixture of independent and constrained refinement
Δρ_max_, Δρ_min_ (e Å^−3^)	0.47, −0.37

Computer programs: *APEX2* and *SAINT* (Bruker, 2016 ▸), *olex2.solve* (Bourhis *et al.*, 2015 ▸), *olex2.refine* (Bourhis *et al.*, 2015 ▸) and *OLEX2* (Dolomanov *et al.*, 2009 ▸).

## supplementary crystallographic information

### Crystal data

**Table:**

C_22_H_17_NO_2_·C_3_H_7_NO	*F*(000) = 848.4030
*M**_r_* = 400.48	*D*_x_ = 1.289 Mg m^−^^3^
Monoclinic, *P*2_1_/*n*	Mo *K*α radiation, λ = 0.71073 Å
*a* = 10.6878 (9) Å	Cell parameters from 4326 reflections
*b* = 8.9088 (7) Å	θ = 3.2–28.1°
*c* = 21.9503 (19) Å	µ = 0.09 mm^−^^1^
β = 99.049 (3)°	*T* = 100 K
*V* = 2064.0 (3) Å^3^	Block, colourless
*Z* = 4	0.36 × 0.28 × 0.16 mm

### Data collection

**Table:**

Bruker APEXII CCD diffractometer	2477 reflections with *I*≥ 2u(*I*)
φ and ω scans	*R*_int_ = 0.139
Absorption correction: multi-scan (*SADABS*; Bruker, 2016)	θ_max_ = 25.1°, θ_min_ = 3.0°
*T*_min_ = 0.368, *T*_max_ = 0.746	*h* = −14→14
31593 measured reflections	*k* = −11→11
3668 independent reflections	*l* = −29→29

### Refinement

**Table:**

Refinement on *F*^2^	41 constraints
Least-squares matrix: full	Primary atom site location: iterative
*R*[*F*^2^ > 2σ(*F*^2^)] = 0.057	H atoms treated by a mixture of independent and constrained refinement
*wR*(*F*^2^) = 0.184	*w* = 1/[σ^2^(*F*_o_^2^) + (0.0846*P*)^2^ + 0.3653*P*] where *P* = (*F*_o_^2^ + 2*F*_c_^2^)/3
*S* = 1.12	(Δ/σ)_max_ < 0.001
3668 reflections	Δρ_max_ = 0.47 e Å^−^^3^
278 parameters	Δρ_min_ = −0.37 e Å^−^^3^
0 restraints	

### Fractional atomic coordinates and isotropic or equivalent isotropic displacement parameters (Å^2^)

**Table:**

	*x*	*y*	*z*	*U*_iso_*/*U*_eq_	
O1	0.73134 (18)	0.7445 (2)	0.43603 (9)	0.0355 (5)	
O2	0.88916 (17)	0.7377 (2)	0.38033 (9)	0.0323 (5)	
O3	0.83886 (19)	0.9615 (2)	0.50335 (9)	0.0400 (5)	
N1	0.49557 (19)	0.2754 (2)	0.23347 (11)	0.0271 (5)	
H1a	0.52980 (19)	0.2410 (2)	0.20230 (11)	0.0326 (7)*	
N2	0.8603 (2)	1.1906 (2)	0.46049 (11)	0.0319 (6)	
C1	0.7855 (2)	0.6922 (3)	0.39023 (13)	0.0271 (6)	
C2	0.7105 (2)	0.5787 (3)	0.35193 (12)	0.0239 (6)	
C3	0.7566 (2)	0.5171 (3)	0.30121 (12)	0.0262 (6)	
H3	0.8380 (2)	0.5456 (3)	0.29315 (12)	0.0314 (7)*	
C4	0.6864 (2)	0.4164 (3)	0.26299 (13)	0.0270 (6)	
H4	0.7197 (2)	0.3763 (3)	0.22878 (13)	0.0324 (8)*	
C5	0.5653 (2)	0.3715 (3)	0.27377 (12)	0.0242 (6)	
C6	0.5206 (2)	0.4299 (3)	0.32576 (12)	0.0263 (6)	
H6	0.4407 (2)	0.3988 (3)	0.33498 (12)	0.0316 (7)*	
C7	0.5920 (2)	0.5320 (3)	0.36341 (12)	0.0251 (6)	
H7	0.5597 (2)	0.5715 (3)	0.39801 (12)	0.0302 (7)*	
C8	0.3682 (2)	0.2265 (3)	0.23901 (13)	0.0272 (6)	
H8a	0.3697 (2)	0.1682 (3)	0.27751 (13)	0.0327 (8)*	
H8b	0.3128 (2)	0.3149 (3)	0.24074 (13)	0.0327 (8)*	
C9	0.3172 (2)	0.1300 (3)	0.18389 (12)	0.0234 (6)	
C10	0.2375 (2)	0.1918 (3)	0.13285 (12)	0.0248 (6)	
C11	0.1944 (3)	0.3436 (3)	0.13080 (14)	0.0344 (7)	
H11	0.2224 (3)	0.4081 (3)	0.16466 (14)	0.0412 (9)*	
C12	0.1145 (3)	0.3979 (4)	0.08172 (16)	0.0452 (8)	
H12	0.0856 (3)	0.4987 (4)	0.08225 (16)	0.0543 (10)*	
C13	0.0737 (3)	0.3074 (4)	0.03015 (16)	0.0456 (9)	
H13	0.0174 (3)	0.3471 (4)	−0.00384 (16)	0.0548 (10)*	
C14	0.1141 (3)	0.1640 (4)	0.02867 (14)	0.0372 (7)	
H14	0.0880 (3)	0.1048 (4)	−0.00706 (14)	0.0447 (9)*	
C15	0.1955 (2)	0.0998 (3)	0.07979 (12)	0.0282 (6)	
C16	0.2339 (2)	−0.0494 (3)	0.07998 (13)	0.0289 (7)	
H16	0.2064 (2)	−0.1097 (3)	0.04472 (13)	0.0347 (8)*	
C17	0.3112 (2)	−0.1129 (3)	0.13030 (12)	0.0266 (6)	
C18	0.3469 (3)	−0.2670 (3)	0.13064 (15)	0.0350 (7)	
H18	0.3170 (3)	−0.3283 (3)	0.09599 (15)	0.0420 (9)*	
C19	0.4230 (3)	−0.3272 (3)	0.17973 (16)	0.0407 (8)	
H19	0.4447 (3)	−0.4306 (3)	0.17957 (16)	0.0489 (10)*	
C20	0.4701 (3)	−0.2375 (3)	0.23099 (15)	0.0368 (7)	
H20	0.5252 (3)	−0.2804 (3)	0.26469 (15)	0.0441 (9)*	
C21	0.4378 (2)	−0.0896 (3)	0.23285 (13)	0.0306 (7)	
H21	0.4707 (2)	−0.0312 (3)	0.26793 (13)	0.0367 (8)*	
C22	0.3555 (2)	−0.0209 (3)	0.18322 (12)	0.0235 (6)	
C23	0.8782 (3)	1.0922 (3)	0.50634 (14)	0.0318 (7)	
H23	0.9249 (3)	1.1247 (3)	0.54448 (14)	0.0381 (8)*	
C24	0.9129 (3)	1.3401 (3)	0.46883 (15)	0.0401 (8)	
H24a	0.8440 (3)	1.4139 (3)	0.4638 (9)	0.0602 (12)*	
H24b	0.9620 (16)	1.3491 (7)	0.5103 (3)	0.0602 (12)*	
H24c	0.9684 (15)	1.3586 (9)	0.4381 (6)	0.0602 (12)*	
C25	0.7882 (3)	1.1510 (4)	0.40096 (14)	0.0445 (8)	
H25a	0.8462 (3)	1.136 (2)	0.3711 (3)	0.0668 (12)*	
H25b	0.7411 (15)	1.0580 (13)	0.4049 (2)	0.0668 (12)*	
H25c	0.7286 (14)	1.2320 (11)	0.3868 (5)	0.0668 (12)*	
H1	0.776 (4)	0.836 (4)	0.4571 (18)	0.085 (13)*	

### Atomic displacement parameters (Å^2^)

**Table:**

	*U*^11^	*U*^22^	*U*^33^	*U*^12^	*U*^13^	*U*^23^
O1	0.0360 (12)	0.0387 (12)	0.0343 (12)	−0.0078 (9)	0.0132 (9)	−0.0087 (10)
O2	0.0293 (11)	0.0377 (11)	0.0307 (11)	−0.0076 (8)	0.0071 (9)	−0.0032 (9)
O3	0.0511 (13)	0.0329 (12)	0.0356 (13)	−0.0006 (9)	0.0058 (10)	−0.0041 (10)
N1	0.0206 (11)	0.0305 (12)	0.0311 (13)	−0.0044 (9)	0.0066 (10)	−0.0092 (10)
N2	0.0321 (13)	0.0264 (12)	0.0366 (15)	0.0015 (10)	0.0039 (11)	−0.0002 (11)
C1	0.0242 (15)	0.0293 (15)	0.0288 (16)	0.0008 (11)	0.0071 (12)	0.0040 (12)
C2	0.0220 (13)	0.0238 (13)	0.0260 (15)	0.0027 (10)	0.0043 (11)	0.0031 (11)
C3	0.0201 (13)	0.0254 (14)	0.0331 (16)	−0.0019 (10)	0.0045 (11)	0.0012 (12)
C4	0.0240 (14)	0.0272 (14)	0.0311 (16)	0.0007 (11)	0.0088 (11)	−0.0040 (12)
C5	0.0215 (13)	0.0230 (13)	0.0278 (15)	0.0016 (10)	0.0030 (11)	0.0018 (12)
C6	0.0199 (13)	0.0284 (14)	0.0316 (16)	0.0005 (11)	0.0070 (11)	0.0012 (12)
C7	0.0230 (14)	0.0275 (14)	0.0255 (15)	0.0017 (11)	0.0055 (11)	−0.0011 (12)
C8	0.0197 (14)	0.0317 (15)	0.0309 (16)	−0.0039 (11)	0.0060 (11)	−0.0039 (12)
C9	0.0166 (13)	0.0278 (14)	0.0267 (15)	−0.0037 (10)	0.0064 (11)	−0.0007 (12)
C10	0.0179 (13)	0.0265 (14)	0.0311 (16)	−0.0033 (10)	0.0075 (11)	0.0036 (12)
C11	0.0327 (16)	0.0330 (16)	0.0387 (18)	0.0027 (12)	0.0097 (13)	0.0042 (14)
C12	0.0374 (18)	0.0412 (18)	0.057 (2)	0.0063 (14)	0.0066 (16)	0.0160 (17)
C13	0.0301 (17)	0.058 (2)	0.047 (2)	0.0023 (15)	−0.0001 (15)	0.0250 (17)
C14	0.0266 (15)	0.0536 (19)	0.0307 (17)	−0.0107 (14)	0.0021 (13)	0.0099 (15)
C15	0.0212 (14)	0.0355 (15)	0.0284 (16)	−0.0060 (11)	0.0055 (11)	0.0037 (13)
C16	0.0240 (14)	0.0356 (16)	0.0281 (16)	−0.0089 (11)	0.0066 (12)	−0.0044 (13)
C17	0.0209 (13)	0.0284 (14)	0.0326 (16)	−0.0046 (11)	0.0106 (12)	−0.0013 (12)
C18	0.0336 (16)	0.0291 (15)	0.045 (2)	−0.0057 (12)	0.0159 (14)	−0.0033 (14)
C19	0.0361 (17)	0.0262 (15)	0.062 (2)	0.0008 (13)	0.0139 (16)	0.0051 (15)
C20	0.0260 (15)	0.0352 (16)	0.048 (2)	0.0010 (12)	0.0036 (14)	0.0132 (15)
C21	0.0217 (14)	0.0340 (15)	0.0358 (17)	−0.0047 (11)	0.0040 (12)	0.0039 (13)
C22	0.0168 (13)	0.0256 (13)	0.0290 (15)	−0.0029 (10)	0.0065 (11)	0.0027 (12)
C23	0.0307 (15)	0.0281 (15)	0.0360 (18)	0.0041 (12)	0.0036 (13)	−0.0066 (13)
C24	0.0380 (18)	0.0304 (16)	0.053 (2)	−0.0011 (13)	0.0099 (15)	0.0012 (15)
C25	0.047 (2)	0.049 (2)	0.0341 (18)	−0.0011 (15)	−0.0030 (15)	0.0005 (15)

### Geometric parameters (Å, º)

**Table:**

O1—C1	1.322 (3)	C11—H11	0.9500
O1—H1	1.02 (4)	C11—C12	1.354 (4)
O2—C1	1.230 (3)	C12—H12	0.9500
O3—C23	1.236 (3)	C12—C13	1.402 (5)
N1—H1a	0.8800	C13—H13	0.9500
N1—C5	1.365 (3)	C13—C14	1.351 (4)
N1—C8	1.452 (3)	C14—H14	0.9500
N2—C23	1.326 (4)	C14—C15	1.427 (4)
N2—C24	1.446 (3)	C15—C16	1.391 (4)
N2—C25	1.452 (4)	C16—H16	0.9500
C1—C2	1.470 (4)	C16—C17	1.392 (4)
C2—C3	1.399 (4)	C17—C18	1.424 (4)
C2—C7	1.393 (3)	C17—C22	1.439 (4)
C3—H3	0.9500	C18—H18	0.9500
C3—C4	1.368 (4)	C18—C19	1.355 (4)
C4—H4	0.9500	C19—H19	0.9500
C4—C5	1.410 (3)	C19—C20	1.407 (4)
C5—C6	1.405 (4)	C20—H20	0.9500
C6—H6	0.9500	C20—C21	1.365 (4)
C6—C7	1.376 (4)	C21—H21	0.9500
C7—H7	0.9500	C21—C22	1.426 (4)
C8—H8a	0.9900	C23—H23	0.9500
C8—H8b	0.9900	C24—H24a	0.9800
C8—C9	1.514 (4)	C24—H24b	0.9800
C9—C10	1.408 (4)	C24—H24c	0.9800
C9—C22	1.406 (3)	C25—H25a	0.9800
C10—C11	1.427 (4)	C25—H25b	0.9800
C10—C15	1.437 (4)	C25—H25c	0.9800
			
H1—O1—C1	114 (2)	H13—C13—C12	119.91 (18)
C5—N1—H1a	118.07 (14)	C14—C13—C12	120.2 (3)
C8—N1—H1a	118.07 (14)	C14—C13—H13	119.91 (19)
C8—N1—C5	123.9 (2)	H14—C14—C13	119.40 (19)
C24—N2—C23	120.4 (2)	C15—C14—C13	121.2 (3)
C25—N2—C23	121.1 (2)	C15—C14—H14	119.40 (18)
C25—N2—C24	118.6 (2)	C14—C15—C10	118.9 (3)
O2—C1—O1	122.2 (3)	C16—C15—C10	119.2 (2)
C2—C1—O1	114.4 (2)	C16—C15—C14	121.9 (3)
C2—C1—O2	123.4 (2)	H16—C16—C15	119.03 (16)
C3—C2—C1	119.8 (2)	C17—C16—C15	121.9 (2)
C7—C2—C1	122.1 (2)	C17—C16—H16	119.03 (16)
C7—C2—C3	118.1 (2)	C18—C17—C16	121.4 (3)
H3—C3—C2	119.41 (15)	C22—C17—C16	119.2 (2)
C4—C3—C2	121.2 (2)	C22—C17—C18	119.4 (2)
C4—C3—H3	119.41 (16)	H18—C18—C17	119.59 (17)
H4—C4—C3	119.59 (16)	C19—C18—C17	120.8 (3)
C5—C4—C3	120.8 (2)	C19—C18—H18	119.59 (17)
C5—C4—H4	119.59 (15)	H19—C19—C18	119.81 (17)
C4—C5—N1	119.4 (2)	C20—C19—C18	120.4 (3)
C6—C5—N1	122.6 (2)	C20—C19—H19	119.81 (17)
C6—C5—C4	118.0 (2)	H20—C20—C19	119.60 (17)
H6—C6—C5	119.79 (15)	C21—C20—C19	120.8 (3)
C7—C6—C5	120.4 (2)	C21—C20—H20	119.60 (18)
C7—C6—H6	119.79 (15)	H21—C21—C20	119.32 (18)
C6—C7—C2	121.5 (2)	C22—C21—C20	121.4 (3)
H7—C7—C2	119.26 (15)	C22—C21—H21	119.32 (16)
H7—C7—C6	119.26 (15)	C17—C22—C9	119.6 (2)
H8a—C8—N1	109.85 (14)	C21—C22—C9	123.2 (2)
H8b—C8—N1	109.85 (14)	C21—C22—C17	117.2 (2)
H8b—C8—H8a	108.3	N2—C23—O3	125.1 (3)
C9—C8—N1	109.2 (2)	H23—C23—O3	117.46 (17)
C9—C8—H8a	109.85 (14)	H23—C23—N2	117.46 (16)
C9—C8—H8b	109.85 (14)	H24a—C24—N2	109.5
C10—C9—C8	120.7 (2)	H24b—C24—N2	109.5
C22—C9—C8	118.8 (2)	H24b—C24—H24a	109.5
C22—C9—C10	120.4 (2)	H24c—C24—N2	109.5
C11—C10—C9	123.2 (2)	H24c—C24—H24a	109.5
C15—C10—C9	119.7 (2)	H24c—C24—H24b	109.5
C15—C10—C11	117.2 (2)	H25a—C25—N2	109.5
H11—C11—C10	119.22 (16)	H25b—C25—N2	109.5
C12—C11—C10	121.6 (3)	H25b—C25—H25a	109.5
C12—C11—H11	119.22 (19)	H25c—C25—N2	109.5
H12—C12—C11	119.52 (19)	H25c—C25—H25a	109.5
C13—C12—C11	121.0 (3)	H25c—C25—H25b	109.5
C13—C12—H12	119.52 (18)		

### Hydrogen-bond geometry (Å, º)

*Cg*5 and *Cg*7 are the centroids of the 10-membered ring system C9–C22 and of the 14-membered anthracene moiety, respectively.

**Table:**

*D*—H···*A*	*D*—H	H···*A*	*D*···*A*	*D*—H···*A*
O1—H1···O3	1.02 (4)	1.59 (4)	2.590 (3)	167 (4)
N1—H1A···O2^i^	0.88 (1)	2.13 (1)	2.973 (3)	160 (1)
C18—H18···O3^ii^	0.95 (1)	2.40 (1)	3.277 (4)	154 (1)
C6—H6···*Cg*7^iii^	0.95	2.80 (1)	3.552 (2)	137 (1)
C7—H7···*Cg*5^iii^	0.95	2.99 (1)	3.646 (3)	138 (1)

Symmetry codes: (i) −*x*+3/2, *y*−1/2, −*z*+1/2; (ii) *x*−1/2, −*y*+1/2, *z*−1/2; (iii) −*x*+1/2, *y*+1/2, −*z*+1/2.

## References

[bb1] Ahmed, A., Faizi, M. S. H., Ahmad, A., Ahmad, M. & Fritsky, I. O. (2020). *Acta Cryst.* E**76**, 62–65. 10.1107/S2056989019016207 PMC6944086 31921453

[bb2] Bourhis, L. J., Dolomanov, O. V., Gildea, R. J., Howard, J. A. K. & Puschmann, H. (2015). *Acta Cryst.* A**71**, 59–75. 10.1107/S2053273314022207 PMC4283469 25537389

[bb3] Bruker (2016). *APEX2*, *SAINT* and *SADABS*. Bruker AXS Inc., Madison, Wisconsin, USA.

[bb4] Bu, J. H., Zheng, Q. Y., Chen, C. F. & Huang, Z. T. (2004). *Org. Lett.* **6**, 3301–3303. 10.1021/ol0487985 15355037

[bb5] Dianu, L. M., Kriza, A., Stanica, N. & Musuc, M. A. (2010). *J. Serb. Chem. Soc.* **75**, 1515–1531.

[bb6] Dolomanov, O. V., Bourhis, L. J., Gildea, R. J., Howard, J. A. K. & Puschmann, H. (2009). *J. Appl. Cryst.* **42**, 339–341.

[bb7] Groom, C. R., Bruno, I. J., Lightfoot, M. P. & Ward, S. C. (2016). *Acta Cryst.* B**72**, 171–179. 10.1107/S2052520616003954 PMC4822653 27048719

[bb8] McKinnon, J. J., Jayatilaka, D. & Spackman, M. A. (2007). *Chem. Commun.* pp. 3814–3816. 10.1039/b704980c 18217656

[bb9] Obali, A. Y. & Ucan, H. I. (2012). *J. Fluoresc.* **22**, 1357–1370. 10.1007/s10895-012-1075-8 22695928

[bb10] Pavitha, P., Prashanth, J., Ramu, G., Ramesh, G., Mamatha, K. & Reddy, B. V. (2017). *J. Mol. Struct.* **1147**, 406–426.

[bb11] Singh, R., Mrozinski, J. & Bharadwaj, P. K. (2014). *Cryst. Growth Des.* **14**, 3623–3633.

[bb12] Spackman, A. M. & Jayatilaka, D. (2009). *CrystEngComm*, **11**, 19–32.

[bb13] Turner, M. J., McKinnon, J. J., Wolff, S. K., Grimwood, D. J., Spackman, P. R., Jayatilaka, D. & Spackman, M. A. (2017). *CrystalExplorer17. University of Western Australia.* http://hirshfeldsurface. net

[bb14] Vidya, V. G. (2016). *Res. J. Recent Sci*, **5**, 41–43.

[bb15] Yan, Y., Chen, J., Zhang, N. N., Wang, M. S., Sun, C., Xing, X. S., Li, R., Xu, J. G., Zheng, F. K. & Guo, G. C. (2016). *Dalton Trans.* **45**, 18074–18078. 10.1039/c6dt03794a 27805194

[bb16] Zhou, Y., Zhou, H., Zhang, J., Zhang, L. & Niu, J. (2012). *Spectrochim. Acta A*, **98**, 14–17. 10.1016/j.saa.2012.08.025 22982382

